# Identification of Differential Gene Expression Pattern in Lens Epithelial Cells Derived from Cataractous and Noncataractous Lenses of Shumiya Cataract Rat

**DOI:** 10.1155/2020/7319590

**Published:** 2020-11-02

**Authors:** Hidetoshi Ishida, Teppei Shibata, Yuka Nakamura, Yasuhito Ishigaki, Dhirendra P. Singh, Hiroshi Sasaki, Eri Kubo

**Affiliations:** ^1^Department of Ophthalmology, Kanazawa Medical University, Ishikawa 9200293, Japan; ^2^Medical Research Institute, Kanazawa Medical University, Kanazawa, Ishikawa 9200293, Japan; ^3^Department of Ophthalmology, University of Nebraska Medical Center, Omaha, Nebraska 68198, USA

## Abstract

The Shumiya cataract rat (SCR) is a model for hereditary cataract. Two-thirds of these rats develop lens opacity within 10-11 weeks. Onset of cataract is attributed to the synergetic effect of lanosterol synthase (Lss) and farnesyl-diphosphate farnesyltransferase 1 (Fdft1) mutant alleles that lead to cholesterol deficiency in the lenses, which in turn adversely affects lens biology including the growth and differentiation of lens epithelial cells (LECs). Nevertheless, the molecular events and changes in gene expression associated with the onset of lens opacity in SCR are poorly understood. In the present study, a microarray-based approach was employed to analyze comparative gene expression changes in LECs isolated from the precataractous and cataractous stages of lenses of 5-week-old SCRs. The changes in gene expression observed in microarray results in the LECs were further validated using real-time reverse transcribed quantitative PCR (RT-qPCR) in 5-, 8-, and 10-week-old SCRs. A mild posterior and cortical opacity was observed in 5-week-old rats. Expressions of approximately 100 genes, including the major intrinsic protein of the lens fiber (*Mip* and Aquaporin 0), deoxyribonuclease II beta (*Dnase2B*), heat shock protein B1 (*HspB1*), and crystallin *γ* (*γCry*) B, C, and F, were found to be significantly downregulated (0.07-0.5-fold) in rat LECs derived from cataract lenses compared to that in noncataractous lenses (control). Thus, our study was aimed at identifying the gene expression patterns during cataract formation in SCRs, which may be responsible for cataractogenesis in SCR. We proposed that cataracts in SCR are associated with reduced expression of these lens genes that have been reported to be related with lens fiber differentiation. Our findings may have wider implications in understanding the effect of cholesterol deficiency and the role of cholesterol-lowering therapeutics on cataractogenesis.

## 1. Introduction

Age-related eye disease is a serious public health issue, and age-related cataract is the leading cause of blindness worldwide [1]. Currently, surgery is the only treatment for cataract. It has been reported that if the progression of cataract is delayed by 10 years, the huge expense associated with surgical intervention can be reduced [2]. Cataracts are caused by the degeneration of the lens protein called crystallin. Lenses have almost no protein turnover and are therefore susceptible to ultraviolet rays, oxidative stress, and glycative stress. These stressors damage protein integrity and function leading to denaturation and aggregation of lens protein, which in turn results in lens opacification [3, 4]. Recent studies have shown that oxidative stress controls various cellular processes associated with cell survival, such as cell proliferation, differentiation, aging, and cell death. It promotes cell apoptosis and senescence and is also associated with many diseases [5, 6]. Oxidative stress and reactive oxygen species (ROS) are a major cause of age-related eye diseases, and diets rich in fruits, vegetables, vitamin C, zeaxanthin, lutein, and multivitamin-mineral supplements are recommended for preventing cataracts and age-related macular degeneration [7–10]. Moreover, several risk factors such as advancing age, genetic predisposition, oxidative stress, and external and internal factors acting adversely on lens homeostasis have been implicated to contribute to the etiology of cataract formation.

Cataracts are suggested to be a multifactorial disease associated with multiple etiological factors. To understand the molecular mechanism underlying cataract formation, animal models are a prerequisite. Shumiya cataract rats (SCRs) have been used as a model to study congenital cataract, exploring the initiation and development of the disease [7–9]. Lens opacity is observed in 66.7% of SCRs [10]. Onset of mature cataract in SCR occurs at around 10-11 weeks of age [10]. The vertebrate lens has a single layer of epithelial cells on its anterior surface. These cells are metabolic engines of lens and are responsible for maintaining its homeostasis and transparency. Any damage to lens epithelial cells (LECs) leads to cataractogenesis. Malfunction and aberrant differentiation of LECs contribute to cataractogenesis in SCRs [11]. Another interesting aspect is that the onset of cataract occurs in the lens epithelium in various types of cataract, such as selenite-induced cataract, UV-exposed cataract (*in vitro*), diabetic cataracts in rats, and noncongenital cataracts in humans [12–16]. The SCR is a model for hereditary cataract, but it also displays the features of oxidative stress-induced cataracts as its onset could be delayed by the administration of antioxidants [7]. Moreover, the genetic basis of cataractogenesis in SCRs is associated with the combination of lanosterol synthase (*Lss*) and farnesyl diphosphate farnesyl transferase 1 (*Fdft1*) mutant alleles in the cholesterol biosynthesis pathway, lowering cholesterol levels in SCR lenses [16]. A previous study showed that inhibition of cholesterol synthesis inhibits cell proliferation in lens epithelial cells (LECs) [17], implicating that adequate cholesterol synthesis is required for maintaining the integrity and function of LECs, which in turn maintains normal lens biology and homeostasis. It has been found that mutations in *Lss* are one of the causes for the onset of human congenital cataracts [18, 19]. Furthermore, lenses treated with lanosterol showed decreased opacity and increased transparency in the case of canine cataracts and reversed the aggregation of crystallin *in vitro* [18]. Hence, SCR is an appropriate model to study the mechanism underlying cataract formation and could help in the development of drugs for the treatment of cataracts.

In this study, we investigated the gene expression changes in LECs of SCRs to analyze the mechanism underlying cataract formation and its association with *Lss* mutation and cholesterol deficiency. A comprehensive analysis of gene expression changes in LECs from SCRs was analyzed using DNA microarray analysis to identify genes associated with cholesterol deficiency for the purpose of screening. Our study may identify the various genes contributing to the onset of congenital cataracts due to cholesterol deficiency. The outcomes of this study could contribute to the development of various therapeutic strategies to treat cataracts.

## 2. Materials and Methods

### 2.1. Animals

All animal experiments were approved by the Committee of Animal Research at Kanazawa Medical University (Permission no. 2017-107) and were conducted in accordance with the Guide for the Care and Use of Laboratory Animals implemented by the National Institutes of Health, the recommendations of the ARVO Statement for the Use of Animals in Ophthalmic and Vision Research, and the Institutional Guidelines for Laboratory Animals of Kanazawa Medical University. SCRs (SCR/Sscr: NBRP Rat No: 0823) were obtained from the National BioResource Project-Rat, Kyoto University (Kyoto, Japan).

We used 5-, 8- and 10-week-old SCRs in this study. Since mature cataracts develop in SCR after 11 weeks of age, rats with precataracts and mild to moderate cataracts were selected (5-10 weeks old). All rats were provided ad libitum access to regular or experimental chow (Sankyo Labo Service, Tokyo, Japan). The animals were sacrificed by administration of a lethal dose of CO_2_. Cataract onset in SCR was uniquely regulated by a specific combination of different mutant (*Lss*^l^) and polymorphic alleles (*Lss*^s^) on the Lss locus [16]. The prerequisite for cataract onset was the *Lss*^s^/*Lss*^l^ genotype, which reduced LSS activity below the threshold that is about 12% of normal [16]. Lens opacity in SCR appears spontaneously at 9-11 weeks of age in 2/3 of animals (*Lss*^s^/*Lss*^l^). In 1/3 of SCR (*Lss*^s^/*Lss*^s^), no cataractous changes appeared even after 11 weeks of age. SCRs having Lss^l^/Lss^l^ are embryonically lethal. Therefore, the rats were divided into two groups, i.e., control (*Lss*^s^/*Lss*^s^; Cat-) and cataractous (*Lss*^s^/*Lss*^l^; Cat+). Cat+ and Cat- SCRs were distinguished via PCR using genomic DNA isolated from the tails of 4-week-old rats. The amplified products were then separated on a 15% gel via polyacrylamide gel electrophoresis (PAGE) to detect *Lss* mutation (*Lss*^s^/*Lss*^s^ or *Lss*^s^/Lss^l^) as reported in a previous study of ours [20]. The sequences of primers used to detect *Lss* mutation were as follows: 5′-GCACACTGGACTGTGGCTGG-3′ and 5′-GCCACAGCATTGTAGAGTCGCT-3′.

### 2.2. RNA Extraction

Total RNA from each LEC sample obtained from SCR was extracted using the miRNeasy Micro Kit (Qiagen, Valencia, CA) following the manufacturer's protocol. Since RNA is extremely susceptible to degradation due to the ubiquitous presence of RNAses in the environment, purity and integrity of RNA were examined and validated as previously described [21]. Quality of total RNA was analyzed by evaluating the RNA integrity number (RIN) using Bioanalyzer RNA analysis (Agilent Technologies Japan Ltd., Tokyo, Japan). All RNA samples showed RIN > 9.0. In this study, we used LECs from SCR, because quality of total RNA obtained from whole lens in Cat+ SCR was poor showing RIN < 7.0.

### 2.3. Microarray Analysis

Cataracts as well as *Lss*-mutation-related genes were screened by microarray analysis using LEC samples from SCR as follows. Total RNAs from each LEC sample obtained from 2 eyes of 5-week-old Cat+ SCR with *Lss^s^/Lss^l^* or Cat- SCRs with *Lss^s^/Lss^s^* as the control were used for the microarray analysis (*n* = 1, each). All samples were processed for the microarray analysis as follows: for RNA labeling and hybridization, GeneChip™ WT Pico Reagent Kit (Applied Biosystems, Thermo Fisher Scientific, Tokyo, Japan) and GeneChip® Rat gene 2.0 ST array (Affymetrix, Thermo Fisher Scientific) were used according to the manufacturer's protocol. Washing, scanning of the arrays, and analysis of scanned images were performed according to manufacturer's instructions. Each chip was normalized by dividing the measurement of each gene by the measurement of the specific control or by average intensity in the single array. Normalized data were exported for subsequent analysis. Genes with a normalized ratio > 2.0‐fold or <0.5-fold were selected as significant genes using the GeneSpring software package version 14.9 (Agilent). A Gene Ontology (GO) analysis was conducted using the DAVID database (https://david.ncifcrf.gov/) for the 110 downregulated genes in LECs of SCR Cat+.

### 2.4. Real-Time Reverse Transcriptase-Quantitative PCR (RT-qPCR)

Total RNAs from each LEC sample obtained from 5-, 8-, and 10-week-old Cat+ SCR with *Lss^s^/Lss^l^* and Cat- SCRs with *Lss^s^/Lss^s^* as the control were used for the microarray analysis (*n* = 3, each). To measure the expression of rat deoxyribonuclease II beta (Dnase2B), heat shock protein B1 (*HspB1*), major intrinsic protein of lens (*Mip*), crystallin, gamma D (*CryγD*), heat shock transcription factor 4 (*Hsf4*), lengsin (*Lgsn*), and tudor domain containing 7 (*Tdrd7*) mRNAs, we conducted a relative quantification of mRNA using Prism 7300 (Applied Biosystems, Thermo Fisher Scientific). The comparative Ct method was used for relative quantification of mRNA expression. The PCR amplification was performed with the TaqMan Universal PCR Master Mix. The probe mix containing the primers for *Dnase2B*, *HspB1, γCry,* and *Mip* was obtained from Thermo Fisher Scientific. All reactions were performed in triplicates. Differential expression for each gene was calculated using the comparative CT method using a predeveloped TaqMan Ribosomal RNA Control Reagent VIC probe as an endogenous control (Thermo Fisher Scientific).

### 2.5. Statistical Analysis

The statistical analysis was performed for all experiments using Student's *t*-test and/or one-way analysis of variance (ANOVA), as applicable. The data were presented as the mean ± standard deviation (SD). A significant difference between the control and treatment group was defined as a *p* value < 0.05 for two or more independent experiments.

## 3. Results

### 3.1. Lens Morphology in 5- and 10-Week-Old SCRs

**Figure 1:**
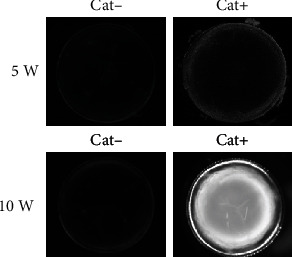
Observation of lens in 5- and 10-week-old Cat- and Cat+ SCRs. Lenses were extracted from 5- and 10-week-old SCRs and placed on glass bottom dishes containing Medium 199 (Thermo Fisher Scientific). Photographs of the lens were acquired and recorded with a stereo microscope.

In Cat- SCR, the lens was clear, at 5 and 10 weeks of age, but in the case of Cat + SCR, a mild posterior and cortical opacity was observed at 5 weeks of age, and severe cortical and nuclear opacity was observed at age 10 weeks (Figure 1).

### 3.2. Analysis of Gene Expression Profile

**Figure 2:**
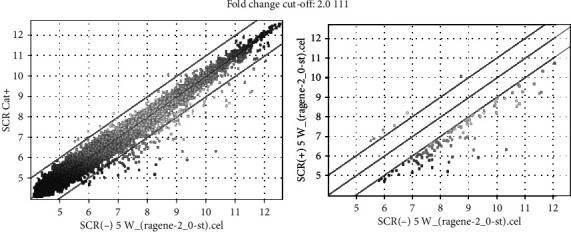
Analysis of gene expression profile. The scatter plots show a representative experiment using lens cDNAs from the Cat- and Cat+ SCRs at 5 weeks. Horizontal and vertical axes represent normal and experimentally generated signals on a logarithmic scale. The *x*-axis represents the log2 fold change, and the dark vertical lines represent cut-offs at 2-fold decrease and increase.

As described in Materials and Methods, LECs from, 5-week-old Cat+ and Cat- SCR were used for microarray analysis (*n* = 1 in each group) to screen genes associated with cholesterol deficiency and cataracts. The data for the microarray analysis was deposited to the Gene Expression Omnibus (GEO) database (Accession number: GSE152616). First, with 28,407 genes on the array, 110 genes were detected that showed a fold change < 0.5 in the Cat+ group compared to those in Cat-, as shown in Figure 2. In the scatter plot, many genes were distributed in the lower part, and specifically, genes whose expression was downregulated could be detected using the scatter plot (Figure 2). Fold changes (<0.5-fold) were observed in 20 top-ranked genes (Table 1) in the microarray analysis including *Dnase2B*, *HspB1, γCry*, *Mip*, beaded filament structural protein 1 (*Bfsp1*), and *Lgsn* that have been reported to be associated with cataract and lens development [22–27]. Additionally, *Hsf4*, *Tdrd7*, gap junction protein epsilon 1 (*Gje1*), beaded filament structural protein 2 (*Bfsp2*), and lens intrinsic membrane protein 2 (*Lim2*) which are also significantly reduced in the SCR lens (<0.5-fold) were linked to human or animal cataracts (Supplement 1) [27–33]. Furthermore, the expressions of six genes were upregulated (2.1- to 2.5-fold) in rat LECs from Cat+ compared to those from Cat-. Of the six genes, five genes were unidentified, and the other was Schlafen 4. However, the function of Schlafen 4 in the lens is not clear.

GO analysis revealed 110 downregulations of genes related to lens development, cellular water homeostasis, negative regulation of apoptotic process, etc. in LECs of Cat+ SCR (Table 2).

### 3.3. Validation of Gene Expression Data Using RT-qPCR

The data from the microarray experiment shown above revealed the downregulation of various genes during the progression of cataracts. Therefore, we selected the following four genes: *Dnase2B*, *Mip*, *HspB1*, *γCry*, *Hsf4*, *Lgsn*, and *Tdrd7* that showed significant changes in expression according to the microarray data, and accordingly, we validated these results using RT-qPCR.

**Figure 3:**
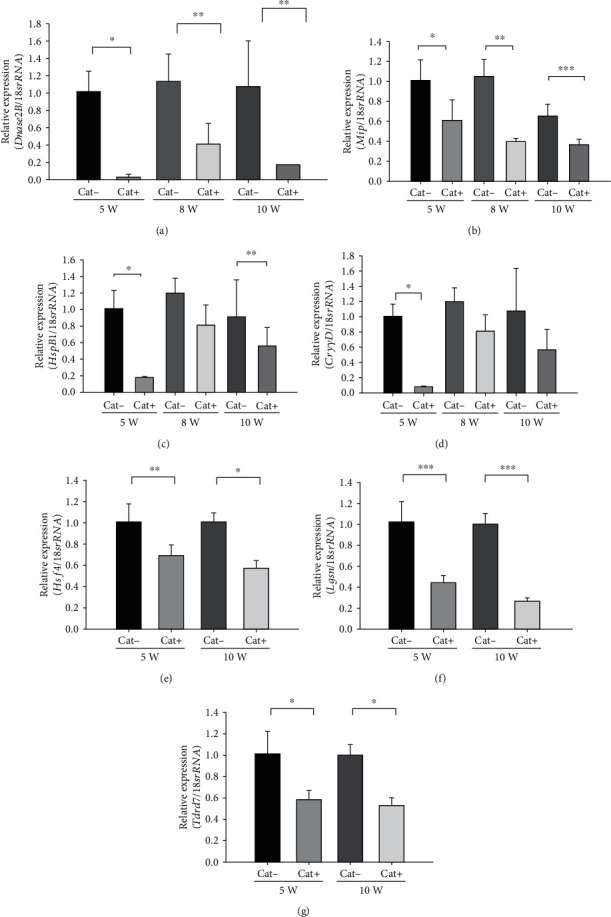
Expressions of *Dnase2B*, *Mip*, *HspB1*, *CryγD*, *Hsf4*, *Lgsn*, and *Tdrd7* mRNA in LECs from Cat- SCR and Cat+ SCR at 5, 8, and 10 weeks of age or 5 and 10 weeks of age. (a) Expression of *Dnase2B* mRNA: ^∗^*p* < 0.02, ^∗∗^*p* < 0.04. (b) Expression of *Mip* mRNA: ^∗^*p* < 0.02, ^∗∗∗^*p* < 0.001, ^∗∗^*p* < 0.04. (c) Expression of *HspB1* mRNA: ^∗^*p* < 0.02, ^∗∗^*p* < 0.04. (d) Expression of *CryγD* mRNA: ^∗^*p* < 0.02, ^∗∗∗^*p* < 0.001, ^∗∗^*p* < 0.04. (e) Expression of *Hsf4* mRNA: ^∗^*p* < 0.02, ^∗∗^*p* < 0.04. (f) Expression of *Lgsn* mRNA: ^∗∗∗^*p* < 0.001. (g) expression of *Tdrd7* mRNA: ^∗^*p* < 0.02. Data are expressed as the mean ± standard deviation (*n* = 3).

Data from the RT-qPCR showed that *Dnase2B* and *Mip* mRNA showed a significant downregulation in 5-, 8-, and 10-week-old Cat+ SCRs compared to Cat- SCRs (Figures 3(a) and 3(b)). The expressions of *HspB1*, *Hsf4*, *Lgsn*, and *Tdrd7* mRNA significantly downregulated in 5- and 10-week-old Cat+ SCRs compared to Cat- SCRs (Figures 3(c), 3(e), 3(f), and 3(g)). However, the expression of *CryγD* mRNA was significantly downregulated only in 5-week-old Cat+ SCRs (Figure 3(d)).

## 4. Discussion

In this study, we comprehensively analyzed gene expression changes in the lens depending on the presence or absence of *Lss* deficiency in SCRs of different ages. SCRs with *Lss* deficiency gradually develop cataracts and show mature cataracts at 11 weeks of age. Lens opacity is slightly observed in 5-week-old SCRs with *Lss* mutations. In microarray analysis, 110 genes were downregulated with 0.5-fold change in Cat+ SCR at 5 weeks of age. It is speculated that carrying the Lss mutation alters many gene expressions in the lens and induces lens opacity. In LECs of Cat+ SCRs with Lss mutations, the expressions of many genes reported to be associated with cataract development such as *Dnase2B*, *HspB1*, *γCry*, *Mip*, *Hsf4*, *Tdrd7*, *Lim2*, *Gje1*, *Bfsp1*, *Bfsp2*, and *Lgsn* were downregulated. In this study, we analyzed *Dnase2B*, *HspB1*, *γCry*, and *Mip* genes using RT-qPCR. We confirmed that expressions of *Dnase2B*, *HspB1*, and *Mip* mRNAs were significantly downregulated in the LECs of Cat+ SCRs with Lss mutation before and after cataract onset. Furthermore, significant downregulation in the expression of *γCry* mRNA was observed in the LECs of Cat+ SCRs with *Lss^s^/Lss^l^* only before the onset of the disease.

In this study, *Hsf4* and *Tdrd7* are the transcription factors (TF) that are found to be reduced in LECs of Cat+ SCR. It has been reported that *Hsf4* mutation causes human congenital and age-related cataracts [28, 29]. Furthermore, the mutations of *Tdrd7* also cause human congenital and age-related cataracts [30, 31]. *Hsf4*- knockout mice (*Hsf4*^−/−^) and *Tdrd7*-homozygous KO mice (*Tdrd7*^−/−^) also cause cataracts [32, 33]. Among 111 genes whose expression was decreased by LECs of Cat+ SCR, genes whose expression was also changed by the lens of *Hsf4*-conditional knockout mice (*Hsf4*-CKO) or *Tdrd7*-homozygous KO mice (*Tdrd7*^−/−^) were analyzed using the iSyTE database (PMID: 29036527 and PMID: 32420594, respectively). Genes whose expressions were commonly reduced in LEC of Cat+ SCR and the lens of *Hsf4*-cKO were *HspB1*, *Sh3bgr*, *Hmox1*, *Atp8a2*, *Pbld1*, *Bfsp1*, *Tdrd7*, and *Dnase2B* (fold change < 0.5) (Supplement 2). A gene whose expression was commonly reduced in LEC of Cat+ SCR and lenses of *Tdrd7*^−/−^ was *HspB1* (fold change < 0.5). Thus, *HspB1* may induce the reduction of *Hsf4* and/or *Tdrd7* as TF in Cat+ SCR. Reduction of *HspB1*, *Sh3bgr*, *Hmox1*, *Atp8a2*, *Pbld1*, *Bfsp1*, and *Dnase2B* genes may be due to the downstream change by the *Hsf4* regulation in Cat+ SCR.

HspB1 also known as heat shock protein 27 (Hsp27) is a protein that is encoded by the *HspB1* gene in humans. HspB1 is an ATP-independent molecular chaperone with a conserved *α*B-crystalline domain in the C-terminus region [34]. Heat shock proteins (Hsp) play a central role in maintaining cellular homeostasis and altering protein folding, thereby protecting *α*A- and other crystalline proteins [35]. Further investigation of Hsp27 revealed that the protein responds to cellular oxidative and chemical stress conditions other than heat shock [35]. In the presence of oxidative stress, Hsp27 plays a role as an antioxidant, decreasing the ROS by raising levels of intracellular glutathione (GSH) [36, 37]. It has been reported that mutation in *HspB1* and/or *α*B-crystallin is responsible for the development of cataract [38] and is considered as major targets for the development of anticataract drugs [39]. We have previously reported that expression of Prdx6, an antioxidant protein, is decreased resulting in an increase in ROS in the LECs of Cat+ SCRs [7]. Thus, low activity of Lss may be involved in the decreased expression of antioxidant genes, causing oxidative stress and inducing cataracts in SCRs.

In mice, DNase II-like acid DNase (*DLAD*; *Dnase2B*) has been identified as a DNA-degrading enzyme that functions during lens enucleation [25]. Since the optimum pH for DLAD is acidic, it was suggested that the nucleus could be engulfed by lysosomes through autophagy and denucleated by the action of DLAD [25]. The lens consists of LECs and differentiated lens fiber cells. In the process of fiber differentiation, intracellular structures such as the nucleus, mitochondria, and endoplasmic reticulum disappear and the lens becomes transparent. In the process of enucleation, the DNA that encodes the genetic information is degraded. For this enucleation process, DLAD plays an important role. In *Dlad* knockout mice, the eye lens seemed to have developed normally; however, undegraded DNA was observed in the lens fiber that contributed to lens opacity. Thus, DLAD is necessary to maintain lens transparency and normal fiber differentiation. *Lss* mutation downregulates the expression of *Dlad* (*Dnase2B*), which could be attributed to cataract development in SCRs.

MIP is a lens fiber major intrinsic protein, also known as Aquaporin 0, which is a water channel in lens fiber cells, facilitating the movement of water, gap junction channels, and solute transporters [40]. MIP plays a crucial role in regulating the osmolarity and homeostasis of the lens and stabilizing cell junctions in the lens nucleus. Currently, 12 mutations in *MIP* have been linked to autosomal-dominant cataracts in humans [26]. The decreased expression of *Mip* in LEC of Cat+ SCRs may disrupt cellular water homeostasis and induce lens fiber swelling and vacuole formation observed in SCR lenses.

The nuclear region of the eye lens is particularly rich in the *γ*Cry protein, which is necessary to maintain structural and functional properties in the lens. *γCry* mRNA was downregulated in the LECs of Cat+ SCRs before the onset of lens opacity compared to Cat- rats. However, there was no significant change in expression of this gene after the onset of cataracts. It is not clear why *γCry* mRNA expression did not decrease in cataractous lenses in SCRs. Further studies are required to understand this phenomenon.

Sterol, such as cholesterol in animals, is a compound that is important as a biosynthetic raw material for steroid hormones, vitamin D, and bile acids, in addition to its role in regulating membrane fluidity as a component of eukaryotic biological membranes [41]. Cholesterol synthesis is carried out through a process of approximately 30 enzymatic reactions using acetyl-CoA as a starting substrate. Lanosterol is the first sterol in the cholesterol synthesis pathway and LSS is an essential rate-limiting enzyme that functions as a downstream element in the lanosterol biosynthetic pathway, catalysing the cyclization of the linear 2,3-monoepoxysqualene to cyclic lanosterol [42]. Congenital cataracts with homozygous and heterozygous *Lss* mutations have been reported to affect the catalysing functions of Lss [18, 19]. Furthermore, the polymorphism rs2968 of the *Lss* gene was associated with nuclear type of age-related cataract (ARC) risk in the Chinese population [43]. Consequently, it has been reported that the mRNA expression of *Lss* was significantly lower in LECs of all subtypes of the ARC group than the control group [43]. Epidemiological studies have shown that individuals receiving statins, which are cholesterol synthesis inhibitors, have an increased risk of being diagnosed with cataracts [44, 45]. Previous studies have reported that Lss might play a significant role in oxidative stress and maintenance of lens transparency [46]. These results indicate that Lss deficiency may be a risk factor of ARC. Additionally, it has also been reported that lanosterol plays a preventive role in cataract formation, inhibiting lens opacity and reversing crystalline aggregation [18]. Additionally, intravitreal injection of lanosterol nanoparticles has been reported to rescue the early stage of lens damage in SCRs [8]. Thus, synthesis of cholesterol by LSS is important to maintain lens transparency.

## 5. Conclusions

In conclusion, our study demonstrated that *Lss* mutations in SCRs result in reduction of Hsf4 and Tdrd7 inducing the downregulation of several genes associated with maintaining lens transparency and identified their relationship with cholesterol deficiency. Cholesterol and Lss in lenses may be important to maintain normal lens homeostasis such as lens fiber differentiation, oxidative and heat stresses, and regulation of lens osmolarity to maintain lens transparency.

## Figures and Tables

**Table 1:** Lists of top 20 genes which are <0.5 downregulated in LECs in 5-week-old Cat+ SCR compared to Cat- SCR.

Gene symbol	Gene description	FC
Lgsn	Lens protein with glutamine synthetase domain	0.064
Clic5	Chloride intracellular channel 5	0.094
Snhg11	Small nucleolar RNA host gene 11	0.124
Crygf	Crystallin, gammaF	0.138
Srd5a2	Steriod-5-alpha-reductase, alpha polypeptide 2	0.165
Cryge| Crygd	Crystallin, gamma E| crystallin, gamma D	0.185
Mip	Major intrinsic protein of lens	0.202
HspB1	Heat shock protein B1	0.204
Crygc	Crystallin, gamma C	0.206
Dnase2B	Deoxyribonuclease II beta	0.207
Lctl	Lactase-like	0.227
Bfsp1	Beaded filament structural protein 1, filensin	0.231
Clic3	Chloride intracellular channel 3	0.260
Fam89a	Family with sequence similarity 89, member 7A	0.272
Cbln2	Cerebellin 2 precursor	0.273
Mall	Mal, T-cell differentiation protein-like	0.281
Wnt7a	Wingless-type MMTV integration site family, member 7A	0.284
Slc24a2	Solute carrier family 24 member 3	0.284
Hmox1	Heme oxygenase 1	0.289
Lpin1	Lipin1	0.302

**Table 2:** GO analysis that showed changes of smaller than 0.5-fold in LECs from 5-week-old SCR Cat+ compared to SCR Cat-.

GO accession	GO term	*p* value
GO:000208	Lens development in camera-type eye	6.18*E*-06
GO:007030	Lens fiber cell development	5.05*E*-04
GO:0034220	Ion transmembrane transport	0.003999
GO:0007601	Visual perception	0.005318
GO:0001654	Eye development	0.005798
GO:0043010	Camera-type eye development	0.021266
GO:0008104	Protein localization	0.030793
GO:0015793	Glycerol transport	0.041241
GO:0009992	Cellular water homeostasis	0.044906
GO:0043627	Response to estrogen	0.051878
GO:0006833	Water transport	0.063030
GO:0009966	Regulation of signal transduction	0.073742
GO:0043066	Negative regulation of apoptotic process	0.078634
GO:0010976	Positive regulation of neuron projection development	0.083444
GO:2001234	Negative regulation of apoptotic signaling pathway	0.091330

## Data Availability

All data generated or analyzed during this study are included in this published article. More details are available from the corresponding author upon reasonable request.
